# Viral Infection in Renal Transplant Recipients

**DOI:** 10.1100/2012/820621

**Published:** 2012-05-02

**Authors:** Jovana Cukuranovic, Sladjana Ugrenovic, Ivan Jovanovic, Milan Visnjic, Vladisav Stefanovic

**Affiliations:** Faculty of Medicine, University of Nis, 18000 Nis, Serbia

## Abstract

Viruses are among the most common causes of opportunistic infection after transplantation. The risk for viral infection is a function of the specific virus encountered, the intensity of immune suppression used to prevent graft rejection, and other host factors governing susceptibility. Although cytomegalovirus is the most common opportunistic pathogen seen in transplant recipients, numerous other viruses have also affected outcomes. In some cases, preventive measures such as pretransplant screening, prophylactic antiviral therapy, or posttransplant viral monitoring may limit the impact of these infections. Recent advances in laboratory monitoring and antiviral therapy have improved outcomes. Studies of viral latency, reactivation, and the cellular effects of viral infection will provide clues for future strategies in prevention and treatment of viral infections. This paper will summarize the major viral infections seen following transplant and discuss strategies for prevention and management of these potential pathogens.

## 1. Introduction

Solid organ transplantation is a therapeutic option for many human diseases. Liver, kidney, heart, and lung transplantations have become standard therapy for selected end-stage diseases. However, complications such as infection and allograft rejection, which are related by immunosuppressive therapy, remain major causes of morbidity and mortality following solid organ transplantation. Epidemiologically, some viral infections are the result of community exposures (influenza, adenovirus), whereas some are commonly transmitted with the allograft (cytomegalovirus, Epstein-Barr virus), and others are the result of more distant exposures reactivated in the setting of immune suppression (chicken pox and varicella zoster as shingles) [1–3]. Multiple simultaneous infections, viral and nonviral, are also common, such as cytomegalovirus (CMV) and human herpes virus 6 or cytomegalovirus and pneumocystis [3–6]. 

The effects of viral infection are classified as direct and indirect. Fever and neutropenia syndrome and invasive disease such as pneumonia, enteritis, meningitis, or encephalitis are considered direct effects. Indirect effects are due to release of cytokines, chemokines, and growth factors in response to viral infection of the body, which deepen immunosuppression and increase risk of other opportunistic infections [2, 7–10]. In addition, viral infection may alter expression of surface antigens (e.g., histocompatibility antigens), provoking graft rejection and/or causing dysregulated cellular proliferation (contributing to oncogenesis). Multiple observational studies implicate infection with HHV 6 and/or HHV 7 as risk factors for CMV disease and CMV infection may trigger HHV 6 and HHV 7 reactivation [3–5, 11, 12]. Recently, coinfection of polyoma virus and CMV has been reported in kidney transplant recipients. Polyoma virus may induce CMV gene expression by stimulating cellular regulator proteins or by its own gene regulator proteins [13–15]. Increased viral replication and persistence may contribute to allograft injury (fibrosis) or chronic rejection. Virus-specific T cells cross-reactive with allo-antigens can alter the memory allo-specific T cell pool and may modulate allograft survival and transplantation tolerance. Viral infection can also lead to the generation of cross-reactive T cells directed against shared antigens between virus and graft (“molecular mimicry”), or neoantigens generated by viral expression within the allograft environment [16–19].

Many viral infections after renal transplantation result from reactivation of “latent” viral infection in the host or from the graft. Whether the virus “awakes” depends on the nature of the virus, the tissue infected, and host immune response. Some latent viruses are metabolically inactive, whereas others are constantly replicating at low levels determined by the effectiveness of the host's immune response. Multiple factors contribute to viral activation after transplantation, including immune suppression (especially reduction of cytotoxic immunity), graft rejection therapy, inflammation (cytokines), and tissue injury. The host response is also less effective because of the mismatch in major histocompatibility antigens between the organ donor and host, which reduces the efficacy of direct pathway antiviral cellular immune responses. These factors render the allograft susceptible to invasive viral infection.

The optimal approach to infection in the solid organ transplant recipient is prevention; failing this, its prompt and aggressive diagnosis and therapy are essential. The sources of infectious agents after transplantation include endogenous organisms, the allograft itself, and the environment. An important principle to consider when evaluating solid-organ transplant recipients (and other immunocompromised hosts) for infection is that the usual inflammatory response to an infectious organism may be attenuated due to immunosuppressive therapy and that therefore the signs and symptoms of infections may be blunted and diagnostic techniques may be compromised [1, 20].

Pretransplant screening of potential organ donors and recipients is an essential part of solid organ transplantation. Several guidelines for pretransplant screening have been published recently, including a consensus conference on the immunocompromised patient, the American Society for Transplantation clinical practice guidelines on the evaluation of renal transplant candidates, and the American Society of Transplant Physicians clinical practice guidelines on the evaluation of living renal transplant donors. Pretransplant screening of the donor and recipient affords an opportunity to assess the safety of transplantation, to determine the prophylaxis and preventive strategies utilized after transplant, to detect and fully treat active infection in the potential recipient prior to transplant, to update the vaccination status of the potential recipient, and to educate the patient and family about preventive measures [21, 22].

The generosity and altruism of organ donation have no nationality and no frontiers. Due to this, increases in migration and international travel have also led to a rise in the number of donors from foreign countries. Linked to this is an increased risk of infections from specific areas of the world, which may persist in a latent or chronic form in the donor and may be transmitted during transplantation. Future advances will likely include the increasing use of rapid molecular diagnostic testing, based on genomics, proteomics, and metabolomics (the high-throughput measurement and analysis of metabolites) and possibly additional testing for emerging pathogen [23–26]. This paper focusses on acute and recurrent viral infections that occur after adult renal transplantation (Table 1).

## 2. Cytomegalovirus

Human cytomegalovirus-human herpesvirus 5 (CMV) belongs to order Herpesvirales, family Herpesviridae, subfamily betaherpesvirinae, genus Cytomegalovirus, species Human herpesvirus 5 [27]. Symptomatic CMV infection occurs in 20 to 60% of all transplant recipients and is a significant cause of increased morbidity and mortality in this population [28, 29]. When compared with other organ transplant recipients, renal transplant patients are at lower risk for CMV, in part due to the lower burden of latent virus in the renal allograft. The incidence of CMV in the renal transplant population is estimated to be between 8 and 32 percent [1, 9]. In the renal transplant population, infection can occur acutely or as reactivation of latent virus. In the absence of prophylaxis, acute infection is most likely to occur between the first and third months following transplant, when immune suppression is at its maximum. However, the onset of acute infection has been delayed by the use of prophylactic antivirals in the early posttransplant period; currently CMV typically occurs after the cessation of antiviral prophylaxis, later in the first year [20, 30]. CMV may be transmitted to transplant recipients via infected donor organs or cellular blood products. Three major patterns of CMV transmission are observed in solid organ transplantation recipients: (1) primary infection develops when a CMV-seronegative individual receives cells latently infected with the virus from a seropositive donor, (2) secondary infection or reactivation infection develops when endogenous latent virus is reactivated in a CMV-seropositive individual posttransplantation, and (3) superinfection or reinfection occurs when a seropositive recipient receives latently infected cells from a seropositive donor and the virus that reactivates posttransplantation is of donor origin [1]. The seronegative recipient of an organ from a seropositive donor is at highest risk (without prophylaxis CMV infections hve been reported to occur in 65–88% recipients, 48–60% of wich develop CMV disease) [9, 28, 29].

 Studies of the effects of CMV disease or asymptomatic infection on graft function and the risk of acute rejection have had conflict results. Some studies have shown an association of CMV disease or CMV infection with an increased graft loss [31, 32], while others failed to shown this effect [33, 34]. Sagedal et al. showed that both CMV disease and surprisingly, even asymptomatic CMV infection were independent risk factors for overall mortality beyond 100 days posttransplantation and reduced graft survival [35]. On a pathological level, CMV can replicate in the kidney tissues and cause acute allograft dysfunction which usually improves with ganciclovir treatment and reduction of immunosuppression [36, 37]. Infection with CMV has also been implicated in chronic allograft nephropathy which is the major reason for the loss of renal allografts after the first year after transplantation [38]. CMV disease has been linked to chronic rejection with arterial myointimal thickening, similar to atherosclerotic coronary disease [28, 39]. However, it is still unclear whether the virus itself leads directly to this glomerulonephropathy.

 The presentation of CMV may be variable, ranging from asymptomatic infection (as defined by the presence of active viral replication) to end organ or disseminated involvement. Commonly patients present with symptoms of fever and malaise sometimes associated with leukopenia, thrombocytopenia, gastroenteritis, pneumonitis, hepatitis, or more rarely retinal or central nervous system involvement [30]. The type of immunosuppression can affect the presentation and severity of illness [40, 41].

### 2.1. CMV Prevention

Serologic screening for antibody to CMV should be performed on both donor and recipient before transplant to identify patients at risk for after transplant infection who might benefit from preventive strategies [23]. Two strategies are commonly used for CMV prevention: (1) universal prophylaxis and (2) preemptive therapy. Universal prophylaxis involves giving antiviral therapy to all “at-risk” patients beginning at or immediately after transplant for a defined time period. In preemptive therapy, patients are monitored at regular intervals for early evidence of CMV replication prior to the onset of clinical symptoms by use of a laboratory assay [42]. Patients with early replication are then treated with antiviral therapy to prevent symptomatic disease. Each approach has advantages and disadvantages that must be considered in the context of the patient and the allograft [43]. Preemptive therapy may decrease drug costs and toxicity. However, it requires excellent logistic coordination in order to obtain, receive, and act on results in a timely fashion; this can be difficult if patients live quite some distance from the transplant center. Prophylaxis might have the theoretical advantage of preventing reactivation of other viruses such as HHV 6, and theoretically may be more likely to prevent indirect effects of CMV. CMV resistance has been observed with both strategies. Drugs that have been evaluated for universal prophylaxis include acyclovir, ganciclovir, valacyclovir, valganciclovir, and immune globulin preparations. Based on current data, the optimal preemptive strategy is unknown. Preemptive therapy is well suited for transplant recipients at low or intermediate risk of CMV disease, while prophylaxis may be better suited for those at high risk [44, 45]. Some studies have concluded that preemptive valganciclovir therapy and valacyclovir prophylaxis are equally effective in the prevention of CMV disease after renal transplantation and there was no difference in overall costs [46, 47]. Same conclusions were obtained in studies that used the ganciclovir as antiviral therapy [48]. A meta-analysis of 32 trials (3737 patients) performed to compare outcomes for various prophylactic antivirals for transplant patients at risk for CMV disease demonstrated that prophylaxis decreased CMV disease, CMV infection, and all-cause mortality [49]. This meta-analysis showed that ganciclovir was more effective than acyclovir in preventing CMV disease. Valganciclovir and intravenous ganciclovir were found to be as effective as oral ganciclovir for prophylaxis. However, the use of ganciclovir may be associated with a greater rate of CMV resistance when compared with valganciclovir, at least in the highest risk recipients [50, 51]. The length of prophylactic treatment varies by institution, but generally lasts for a minimum of 3 months.

### 2.2. CMV Diagnosis

Many techniques are currently available to aid in the diagnosis of CMV disease. These include serology, viral culture, shell vial culture, pp65 antigenemia test, and qualitative and quantitative nucleic acid detection assays [52]. Although histopathologic diagnosis remains the gold standard, noninvasive measures of viremia are used more commonly to determine the presence or absence of CMV. The antigenemia assay (detection of lower matrix phosphoprotein pp65 in leukocytes) and nucleic acid testing including CMV-PCR and hybrid-capture DNA are typically used with the choice of methodology varying among centers [20, 28]. A clinically useful test should ideally have good sensitivity and specificity, be able to detect asymptomatic CMV infection and predict CMV disease thus helping in treatment decisions, and be technically easy to perform. Although generally accepted as the most sensitive methodology for CMV detection, nucleic acid testing has not been well-standardized and recent studies have demonstrated substantial interlaboratory variability [53, 54]. Quantitative CMV assays have two prominent gaps: neurologic disease, including chorioretinitis, and gastrointestinal disease, including invasive colitis and gastritis. In these syndromes, the CMV assays are often negative and invasive (biopsy) diagnoses are often necessary.

The schedule for screening should be linked to the individual's risk for infection. In the patient being treated for CMV infection, the assays provide an endpoint (a negative assay) for therapy and the reinitiation of prophylaxis. In the patient at high risk after the completion of prophylaxis, weekly to biweekly screening should be considered to assure the absence of infection for 3 to 6 months [2].

### 2.3. CMV Therapy

Treatment of active CMV disease requires a combination of immunomodulation, antiviral therapy, and reduction of immunosuppression, if possible [30]. The standard of care for treating CMV disease is 2 to 3 weeks of intravenous ganciclovir (5 mg/kg twice daily, dose adjustments for renal dysfunction) with demonstration of clinical and virological responses to therapy [30]. In contrast, oral ganciclovir should not be used for the treatment of CMV disease because of the limited absorption and poor bioavailability. In seronegative patients and those slow to respond to therapy, the addition of CMV hyperimmune globulin (100 to 150 mg/kg per dose intravenously, given monthly) may be useful [2]. More recently, the introduction of valganciclovir has allowed for the oral treatment of CMV disease in SOT recipients. In a trial of 21 renal transplant recipients with symptomatic CMV disease and viremia who were treated with valganciclovir, all cleared their infection and none experienced relapse during a mean follow-up of 5.5 months [55]. Recently, a multicenter randomized control trial was performed with 321 solid organ transplant recipients which demonstrated oral valganciclovir was not inferior to intravenously ganciclovir for the initial treatment of CMV viremia [56]. Indeed, valganciclovir was recently shown to be as effective as intravenous ganciclovir in the treatment of mild-to-moderate (i.e., nonsevere) CMV disease [57]. The duration of treatment for CMV disease should be individualized and guided by virological and clinical surveillance. Undetectable viraemia should be achieved prior to discontinuation of therapy in order to reduce the risk of clinical relapse. Previous studies have shown that persistent viraemia at the end of therapy is associated with a higher risk of disease relapse [58]. Alternative therapies (not Food and Drug Administration-approved for use in solid organ transplant recipients) include foscarnet, cidofovir, and leflunomide; these are reserved for treatment of antiviral resistance. Foscarnet is active against most ganciclovir-resistant strains of CMV but has neurotoxicity and renal toxicity with severe magnesium wasting. Cidofovir has been used in renal transplant recipients, however often with nephrotoxicity. Both foscarnet and cidofovir may exhibit synergistic nephrotoxicity with calcineurin inhibitors [2]. One of the biggest challenges regarding the treatment of CMV is the emergence of antiviral resistance. Although this is more commonly noted in lung and pancreas transplant recipients, CMV resistance to ganciclovir has been observed in renal transplant recipients. Ganciclovir resistance should be suspected when patients have persistent, unchanged viremia and/or symptoms at 2 weeks into therapy and in such cases, genotypic assays for the detection of the mutations associated with antiviral resistance should be performed. Treatment of resistant isolates may include the use of foscarnet with or without ganciclovir, or cidofovir [30]. Small case studies have demonstrated some efficacy of leflunomide to treat CMV disease in renal transplant patients. A prospective study of 17 patients treated with leflunomide for CMV demonstrated viral clearance and healing of infected organs in 15 patients (88%) [58]. Other potential therapeutic agents for multidrug-resistant CMV include immunoglobulins, leflunomide and artesunate although data supporting their use remains anecdotal [59–61]. Hence, there is a need to identify novel agents and strategies for the management of CMV infection and disease.

## 3. Herpes Simplex Virus

Human herpesvirus 1-herpes simplex virus 1 (HSV) belongs to subfamily Alphaherpesvirinae, genus Simplexvirus, species Human herpesvirus 1. Seroprevalence for HSV-1 in the adult population is 60%, while the incidence of HSV in renal transplant recipients is estimated to be approximately 53% [1, 30]. HSV most commonly causes reactivation infection but may cause primary infection, transmitted by person-to-person contact or via the allograft. Following primary infection, the virus remains latent in the sensory nerve ganglia. In the absence of prophylaxis, HSV may be seen early, in the first posttransplant month. Reactivation or primary HSV infection results in oral or genital mucocutaneous lesions, occasionally causes pneumonitis, tracheobronchitis, esophagitis, hepatitis, or disseminated infection [1, 30]. Disease may be more severe, invasive, and prolonged in transplant recipients. HSV esophagitis may present with superficial, punched-out lesions that may be superinfected with Candida. HSV is the most common form of encephalitis in transplant recipients. HSV PCR and cultures from cerebrospinal fluid can assist diagnosis. Diffuse interstitial pneumonitis may complicate disseminated disease. Multivisceral involvement with HSV infection is often fatal [62–64].

Diagnosis may be made with the aid of direct fluorescence antibody for HSV from vesicular lesions or PCR from cerebrospinal fluid or visceral tissue samples. Due to high seroprevalence in the adult population, serologies are rarely helpful in the setting of active infection [20].

Treatment of HSV infection with acyclovir has been shown to be effective. Mucocutaneous HSV infection in solid-organ transplant recipients should be treated with oral acyclovir while disseminated or deep HSV infection should always be treated with intravenous acyclovir [1, 30]. The dose should be adjusted in patients with renal failure. Although more toxic than acyclovir, ganciclovir and foscarnet are also effective against HSV. A concern with respect to chronic acyclovir use is the development of resistant mutants of HSV. Acyclovir resistance may arise from mutations in the genes for thymidine kinase or DNA polymerase. Acyclovir resistance has been rarely reported in some strains; foscarnet, cidofovir, and topical trifluridine may be considered for treatment of resistant virus, although careful monitoring of renal function is required [1, 2, 20, 65].

## 4. Varicella Zoster Virus

Human herpesvirus 3-Varicella zoster (VZV) belongs to subfamily Alphaherpesvirinae, genus Varicellovirus, species Human herpesvirus 3. VZV causes two distinct clinical diseases following transplantation. Ninety percent of adult solid-organ transplant recipients are VZV seropositive pretransplantation, and thus VZV reactivation in this group will cause herpes zoster. The remaining 10% are VZV seronegative and are thus at risk of primary infection. The incidence of VZV in renal transplant recipients is lower than HSV and is approximately 4 to 12% [1]. VZV causes a spectrum of disease in solid organ transplant recipients, ranging from localized dermatomal zoster (involving a few adjoining dermatomes) to multidermatomal or disseminated zoster with or without visceral involvement. In a cohort of 869 adult solid organ transplants (434 renal transplant recipients), 7.4% of the renal transplant recipients had herpes zoster with a median time to onset of 9 month [66]. The main complications of a VZV infection in this immunosuppressed population were disseminated intravascular coagulation (DIC) and hepatitis in almost half and pneumonitis in 29% of patients. Infections of the allograft and of the CNS as well as pancreatitis were also described. Concomitant bacterial or CMV infections have been reported too [67].

Unilateral vesicular lesions in a dermatomal pattern are usually sufficiently characteristic of herpes zoster to enable a clinical diagnosis; however, culture of VZV in susceptible cell culture lines, demonstration of multinucleated giant cells on Tzanck smear, and/or direct immunofluorescence is recommended for confirmation. These techniques can also be used for diagnosis in cases of primary infection [1]. Pretransplant screening for previous VZV infection should be performed and naïve patients should be vaccinated with live attenuated varicella vaccine before transplant whenever possible to avoid primary VZV infection after transplantation, an often severe disease with a high mortality rate [67]. However, due to the fact that the VZV vaccine is a live vaccine, the vaccine should not be given it if transplant is expected within four to six weeks to prevent active viral shedding at the time of transplant [30]. A VZV naïve transplant patient who is exposed to someone infected with varicella should receive varicella immune globulin within 96 hours of exposure (if available). If VZIG is not available, or the patient presents greater than 96 hours following exposure, acyclovir may be considered for postexposure prophylaxis [30]. Posttransplant prophylaxis against reactivation of VZV and also HSV is recommended to prevent severe recurrences and consists of ganciclovir in patients needing CMV prophylaxis. Those patients who do not require CMV prophylaxis can receive valacyclovir or acyclovir for approximately one to three months posttransplant [30]. After transplantation, most authorities defer vaccination with live vaccines; killed vaccine appears to be beneficial [67, 68]. VZ-immunoglobulin is recommended for immunocompromised individuals with exposures to varicella or zoster; protection is incomplete [69].

## 5. Epstein-Barr Virus

Epstein-Barr virus-Human herpesvirus 4 (EBV) belongs to subfamily Gammaherpesvirinae, genus Lymphocryptovirus, species Human herpesvirus 4. It can infect B lymphocytes as well as malignant cells of several lineages, including T lymphocytes, epithelial cells, and smooth muscle cells. EBV is associated with a wide range of malignancies, including posttransplant lymphoproliferative disorder (PTLD), Hodgkin and non-Hodgkin lymphomas, nasopharyngeal carcinoma, gastric carcinoma, and leiomyosarcoma [70]. Nearly every human is infected before adulthood. Infection early in childhood is usually asymptomatic, while delayed primary infection is typically manifested through the symptoms of infectious mononucleosis. Once infection occurs, the viral genome is maintained for life in a small fraction of B lymphocytes. In the setting of allogeneic transplantation when iatrogenic immunosuppression is used to prevent graft rejection, an unintended consequence is failure to suppress active EBV infection, which is accompanied by a heightened risk of developing PTLD [71]. PTLD is a potentially life-threatening neoplasm exhibiting a spectrum of histopathologies ranging from reactive-appearing, polyclonal lymphoid infiltrates to sheets of undifferentiated cells that are morphologically indistinguishable from malignant lymphoma or plasma cell myeloma [70].

The majority of symptomatic infections in renal transplant recipients are primary infection, likely related to reactivation of donor virus. Since ninety percent of adults have antibodies to EBV by age 40, symptomatic infection is most commonly seen in pediatric populations. Renal transplants have the lowest risk of acquiring PTLD in comparison with other transplant populations (approximately 1 to 3%). PTLD most commonly occurs in the first year after transplant [20, 30]. Symptoms are often quite nonspecific, for example, fever, malaise, and anorexia, and some patients are asymptomatic. PTLD frequently presents as a rapidly enlarging mass in the grafted organ, in lymph nodes, filling the marrow space, or in extranodal sites such as upper airway or intestine [72].

Nearly all transplant recipients are infected or eventually become infected by EBV, yet only a fraction will develop PTLD. Risk factors for PTLD are as follows: EBV seronegativity at the time of transplant, active primary EBV infection at the time of transplant, underlying disease leading to transplantation, prior splenectomy, second transplant, patient age (children and older adults), coinfection by cytomegalovirus and other viruses, acute or chronic graft-versus-host disease, immunosuppressive drug regimen and intensity, cytokine polymorphisms, HLA type and extent of HLA mismatch, or the presence of multiple risk factors [70]. Active primary EBV infection is a contraindication to transplantation [73]. The type of immunosuppression can alter the risk of development of PTLD, with higher incidence rates observed in patients receiving cytolytic therapies, including antithymocyte globulin and OKT3 [74]. Fludarabine, azathioprine, and other agents causing profound T-cell suppression or mutagenicity are also implicated in PTLD pathogenesis [75, 76].

PTLD is divided into four major histopathologic subtypes with corresponding clinical and biologic features, as described in the World Health Organization (WHO) subclassification scheme [77]. These include early lesions, polymorphic PTLD, monomorphic PTLD, and classical Hodgkin lymphoma type PTLD. Although these lesions may bear microscopic resemblance to diseases arising sporadically in otherwise healthy individuals (e.g., infectious mononucleosis, diffuse large B-cell lymphoma, myeloma, Hodgkin lymphoma, and age-related B-cell lymphoproliferative disorder), their occurrence in the setting of transplantation warrants a diagnosis of PTLD given that the natural history and recommended therapy for PTLD differ from those for lesions having similar histologic features in nonimmunocompromised hosts. In terms of natural history, PTLD almost always progresses quite rapidly to a fatal conclusion unless promptly recognized and treated [70]. The ability to reduce or eliminate immunosuppressive drugs is a helpful strategy for restoring natural antiviral and antineoplastic immunity.

Biopsy is necessary to confirm a diagnosis of PTLD and to rule out other neoplastic or infectious lesions [73]. Histochemical stains are helpful in narrowing the differential diagnosis. Immunohistochemistry is somewhat less reliable, since viral proteins such as LMP1, LMP2, EBNA1, and EBNA2 may be expressed focally or inconsistently in PTLD cases with EBV infection [78].

EBV load testing is commonly used to assist in diagnosis and monitoring of transplant recipients, despite a paucity of clinical trials demonstrating the utility of EBV DNA measurement in such settings. Indications for EBV load testing in an immunosuppressed transplant recipient typically include lymphadenopathy or other mass lesion, organ dysfunction, fever, malaise, or other signs and symptoms suggestive of PTLD. In addition, routine monitoring of EBV load in high-risk patients can help identify PTLD before signs and symptoms appear [79, 80]. An EBV load above the laboratory's established threshold for PTLD should be conveyed to the clinician immediately so it may trigger a search for putative sites of disease followed by biopsy, when reasonable, to establish a histopathologic diagnosis. Even in the absence of biopsy-diagnosed PTLD, preemptive intervention may be used to resolve laboratory-detected disease [71]. Preemptive therapy may include reducing immunosuppression and infusing anti-CD20 antibody or donor T cells. Patients at high risk for PTLD (e.g., those who are intensely immunosuppressed and who were seronegative at the time of transplant) tend to be monitored frequently (e.g., weekly in the first few months after transplant and then monthly) so that preemptive therapy may be considered [71, 81]. Optimally designed trials should measure EBV load once monthly during the first year, with some patients continuing to be frequently monitored beyond the first year if they have a history of high EBV loads, if their drug regimen is particularly immunosuppressive, or in the aftermath of discontinuing antiviral prophylaxis. The European Best Practice Guidelines for Renal Transplantation recommend using EBV load to gauge intervention [73]. In its practice guidelines, the “Kidney Disease: Improving Global Outcomes” Transplant Work Group recommends that high-risk renal transplant patients be tested for EBV nucleic acid once within the first week after transplant, then at least monthly for 3 to 6 months, and then every 3 months for the rest of the first year. Additional EBV testing is recommended after treatment for acute rejection [82].

Clinical management of PTLD typically involves reducing iatrogenic immunosuppression so that natural immunity against EBV and the neoplastic clone is restored [71, 82]. Complementary interventions include infusing donor lymphocytes, infusing EBV-specific cytotoxic T cells that are grown ex vivo by exposing HLA-matched T cells to EBV antigens [83–85] and infusing anti-CD20 monoclonal antibody (e.g., rituximab) [86–88]. If initial intervention is insufficient, more traditional cancer treatment with radiation and multidrug chemotherapy is used [72].

Antiviral therapy with acyclovir or ganciclovir remains controversial as these agents are not active against the latent form of EBV found in PTLD. Outcomes with PTLD in renal transplant recipients vary depending on the site of involvement. Patients with isolated allograft involvement have a five-year survival of approximately 68% compared with those patients with PTLD extending beyond the transplanted kidney whose five-year survival varied between 36 and 38 percent [89].

## 6. Human Herpesvirus 6, Human Herpesvirus 7, and Human Herpesvirus 8

Human herpesvirus 6 and human herpesvirus 7 (HHV 6 and HHV 7) belong to subfamily Betaherpesvirinae, genus Roseolovirus, species Human herpesvirus 6 and human herpesvirus 7. Both viruses are ubiquitous with a high seroprevalence in adults. After primary infection, these viruses establish a latent or persistent infection that remains for the lifetime of the host. The viruses are lymphotropic, but HHV 6 in particular, which uses the CD46 molecule as its receptor, may also infect other cell types, such as monocytes and epithelial and endothelial cells. HHV-6 isolates are classified into two variants (termed HHV 6A and HHV 6B) on the basis of distinct genetic, antigenic, and biological characteristics. The specific pathogenicity of each variant remains poorly understood [90, 91]. Most infections in renal transplant recipients are due to HHV 6B. HHV7 uses the CD4 molecule as its receptor and is more strictly lymphotropic.The stimulus for reactivation of beta-herpesviruses in renal transplant recipients is an immunosuppressive regimen. In addition, these viruses possess immunomodulating properties, including the ability to alter the expression of immune activation molecules, modulate expression of several cytokines and chemokines and induce apoptosis in lymphocytes, which may contribute to immunosuppression. Productive infection of CD4 T cells results in cytopathic effects and cell destruction [92]. The ubiquitous nature of HHV 6 and HHV 7 creates conditions for the development of concurrent infection and interaction between these viruses. The kinetics of the activation of these viruses during the posttransplantation period suggest that HHV 7 may act as a cofactor for HHV 6 and CMV reactivation, while both HHV 6 and HHV 7 may act as cofactors in the pathogenesis of CMV disease and acute rejection [4, 5, 11]

HHV 6 has been associated with fever, rash, encephalitis, hepatitis, myelosuppression, and interstitial pneumonitis [93]. Diagnoses of HHV 6 and HHV 7 infections are made by qualitative and quantitative molecular assays, by tissue immunohistochemistry, and/or peripheral blood mononuclear cell culture. Treatment is similar to CMV and should involve reduction of immunosuppression and ganciclovir, but cidofovir and foscarnet have also been utilized [11].

Human herpesvirus 8 (HHV 8) belongs to subfamily Gammaherpesvirinae, genus Rhadinovirus, species Human herpesvirus 8. HHV 8 has been associated with Kaposi's Sarcoma, primary effusive lymphoma, and Multicentric Castleman's Disease (lymphoproliferative disorder). Infection in the renal transplant population is thought to most commonly happen through reactivation of latent virus, although primary infection after transplant can occur and can be acquired through the allograft itself [94]. Transplantation-associated Kaposi's Sarcoma occurs in 0.2 to 5% of renal transplant recipients, varying by ethnic group and immunosuppressive regimen [95–97]. Of all of the tumors in solid-organ transplantation, Kaposi's Sarcoma occurs at the shortest interval after transplant. HHV 8 is thought to lead to Kaposi's Sarcoma through upregulation of vascular endothelial growth factor (VEGF) receptor Flk1/KDR in endothelial cells [98]. Therapy for Kaposi's Sarcoma includes reduction of immunosuppression and cytotoxic chemotherapy. Sirolimus (rapamycin), an immunosuppressive drug used in kidney-transplant recipients, probably has antineoplastic effects. The immunosuppressive and antineoplastic effects of sirolimus may be due to a common mechanism [98–101]. It is thought that sirolimus inhibits not only production of VEGF but also dampens its effects on endothelial cells, and it has been suggested that sirolimus inhibits the progression of Kaposi's sarcoma in kidney-transplant recipients while exerting an antirejection effect on organ allografts [98].

## 7. BK and JC Virus

BK polyomavirus (BKV), JC polyomavirus (JCV), and Simian virus belong to family *Polyomaviridae*, genus Polyomavirus. In the last 10 years, improved immunosuppression drugs have decreased the rates of acute rejection in kidney transplantation but have also led to the emergence of polyomavirus-associated nephropathy (PVAN). This occurs in 1% to 10% of patients with kidney transplantation and is caused by BK virus in more than 95% of cases. Less than 5% of cases are attributed to the JC virus. Initially, lack of recognition or late diagnosis of PVAN resulted in rapid loss of graft function in more than 50% of patients [102, 103].

Serological evidence of past BKV exposure has been seen to reach 90% in adolescents and adults around the world and seems not to have changed since the first discovery of BKV in the 1970s. Similar to earlier studies, Hirsch et al. [104] found 80% seropositivity in a prospective study of patients with kidney transplantation. Accordingly, mismatched BKV serostatus between donor graft and recipient is significantly less frequent than cytomegalovirus mismatches and, in the absence of change, cannot explain the emergence of PVAN in the last decade. However, higher BKV antibody titers in kidney donors and lower titers in recipients have been identified as risk factors for BKV replication and viremia posttransplantation [102]. The recent increase in BK infections has been attributed, in part, to the use of more potent immunosuppressive regimens. However, no specific immunosuppressant medication or combination has been demonstrated to increase the risk of nephropathy. Caucasian race, cadaveric renal transplant, older age, presence of diabetes, and combined kidney and pancreas transplants have all been shown to be associated with BK virus nephropathy [105].

Primary infections with BKV and JCV are typically subclinical or linked to mild respiratory illness and are followed by viral dissemination to the sites of lifelong persistent infection. The major sites of persistence for both BKV and JCV are the cells of the kidney and urinary tract [106]. BKV DNA has been found in 30 to 50% of normal kidney tissues, with distribution patterns of small foci throughout the cortex and medulla, and in 40% of ureters. JCV DNA can be detected in approximately 10 to 50% of normal kidney samples [106]. Reactivation of latent virus has been reported in old age, pregnancy, and diabetes mellitus, and immunosuppression associated with congenital immunodeficiency, organ transplantation, or HIV infection. The most striking feature of BK infection in kidney transplant recipients is the lack of fever, malaise, myalgias, leukopenia, anemia, thrombocytopenia, or other symptoms or signs typical of viral infection, despite viral loads exceeding a billion copies/mL in the urine or 100 000 copies/mL in the blood [107]. BKV nephropathy presents with renal dysfunction without other clinical signs or symptoms.

Although JC virus has been rarely implicated as a cause of nephropathy either alone or in combination with BK, it is more commonly seen as a cause of Progressive Multifocal Leukoencephalopathy, a demyelinating process involving the cerebral white matter. Presenting symptoms include progressive neurologic impairment, which can progress to dementia [108].

Laboratory monitoring strategies for BKV are still evolving. Quantitative nucleic acid-based viral load assay of urine or blood are becoming widely used for BKV screening [107]. Detectable virus in the blood is more predictive of BKVN than viruria alone. Some medical centers prefer urine cytology as the primary screening technique [109]. While urinary “decoy cells” have excellent sensitivity for the detection of overt BKVN, polymerase chain reaction (PCR) is four times more sensitive than urine cytology for monitoring asymptomatic viruria [110]. Additionally, PCR provides a more objective estimate of true viral load and can distinguish BK viruria from JC viruria. JCV excretion in the urine is usually insignificant, although very rare cases of JCV associated interstitial nephritis are on record. Decoy cells are not stable, whereas DNA is, and PCR may be used for monitoring of patients at a distance from the transplant center. The relative costs of PCR versus cytology are a center-dependent variable. Laboratory screening for BKV should certainly be done for any unexplained rise in serum creatinine [107]. The cost of screening was found to be substantially offset by the savings related to reductions in immunosuppression following diagnosis of BKVN. No anti-viral agents were administered. Definitive diagnosis of BKVN requires a biopsy and demonstration of BKV inclusions in tubular epithelial or Bowman's capsular epithelial cells. Viral infection is accompanied by varying degrees of inflammatory cell infiltrates, tubular atrophy, and fibrosis. The cytopathic effect seen by light microscopy is typical, but not pathognomonic for BKVN. Confirmatory immunohistochemistry or in situ hybridization studies are usually performed using antibodies against specific for BKV proteins or probes complementary to viral DNA [103, 105, 107]. Electron microscopy can be used to demonstrate unenveloped, viral particles, approximately 40 nm in diameter. Since BKVN can be focal in distribution, ideally two biopsy cores should be examined. The availability of medullary parenchyma increases the diagnostic sensitivity. Negative biopsy results cannot rule out BKVN with certainty, and a diagnosis of “presumptive BKVN” can be made if there is renal allograft dysfunction associated with BK viremia [107]. A definitive diagnosis of rejection concurrent with viral nephropathy should only be made if there is endarteritis, fibrinoid arterial necrosis, glomerulitis, or accumulation of the complement degradation product C4d along peritubular capillaries.

BK and JC shedding have been reported to coexist in 16% of patients with decoy cells with the long-term prognosis of this finding being similar to that of patients with pure BK shedding. Only a few published cases of PVAN have been attributed to JC virus. In a cohort of patients screened for decoy cells, 27% were found to have JC (only) shedding in the urine. These patients were older and showed decoy cells appearing later compared with patients with pure BK shedding [111]. Among these were six patients with JCV-associated nephropathy, documented by positive cytology, positive plasma quantitative PCR, and positive SV40 staining. With decreased immunosuppression, many of these patients continued to shed JCV. They all had stable serum creatinin, and the only indication for biopsy was the presence of “decoy” cells in the urine. These patients did not have graft dysfunction or loss. Progressive multifocal leukoencephalopathy, a complication of JCV infection typically found in patients with HIV infection, has rarely been reported in renal transplant recipients [109].

Reduction/adjustment in immunosuppression remains the cornerstone for the treatment or prevention of PVAN [102–105]. Because the reconstitution of the immune system with control of the infection takes 4 to 12 weeks, it is imperative to start treatment as early as possible [112]. The one risk encountered with reduction in immunosuppression is the development of acute rejection. The preliminary results of Wali et al. [113] reflect the protocol used at the University of Maryland, which consists of intensive screening with subsequent stepwise decrease in immunosuppression. This protocol has resulted in clearance of Vm with no graft loss or significant rejection diagnosed. Specifically immunosuppression reduction is as follows: step 1, decrease in the dose of MMF by 50% immediately after diagnosis; step 2, 50% decrease in the target trough level of tacrolimus at 3 months if decoy cells persisted; and step 3, elimination of MMF at 6 months if decoy cells persisted. Maintenance dual therapy consisted of the modified dose of tacrolimus and maintenance dose of prednisone (not exceeding 7.5 to 15 mg per week). In addition to decrease in immunosuppression, several centers have reported the use of several antipolyomaviral agents, with anti-BKV activity in vitro. These include cidofovir, leflunomide, quinolones, and intravenous immunoglobulin [102, 105, 114]. None of these agents have been approved by the Food and Drug Administration for PVAN treatment. However, they have been used empirically, and in many cases at advanced stages of the disease when graft dysfunction persists or worsens. The efficacy of these antiviral agents is difficult to determine, as they have been used in combination with reduction in immunosuppression, and even, in certain cases, in combination with each other. In addition, no prospective, randomized control trials have been conducted. In summary, examination of the available literature demonstrates that none of the ancillary treatments described has been conclusively proven to be efficacious. Most studies were neither randomized nor double-blinded, and histologic grading of PVAN was often missing. In addition, in many cases, renal dysfunction was present at the time of diagnosis. Multicenter prospective studies are needed to clarify this important issue stratifying histologic grading, renal function, viral load diagnosis, and most importantly, an evaluation of different strategies assessed independently.

Early diagnosis with close monitoring of renal function andserial determinations of Vm continue to represent the most efficacious tool to control PVAN. Systematic reduction in immunosuppression has not been associated with clear evidence of increased chronic rejection but longer times of followup and more stringent studies are necessary to determine the longterm impact of the interventions for PVAN on long-term graft outcomes.

## 8. Hepatitis B and C Virus

Hepatitis B virus (HBV) belong to family *Hepadnaviridae*, genus Orthohepadnavirus,species Hepatitis B virus. In patients with chronic kidney disease undergoing renal transplantation, despite improved outcomes, liver disease has persisted as an important cause of morbidity and mortality. The prevalence of hepatitis B virus infection among patients on renal replacement therapy has decreased because of the implementation of preventive strategies in the dialysis population [115, 116]. Currently, it is estimated the prevalence in the hepatitis disease population ranges from 0.1 to 0.4 percent [30]. Approximately 2 to 10 percent of patients with a history of Hepatitis B before transplantation will reactivate posttransplant. The most important factor influencing HBsAg seropositive status in recipients is the acquisition of HBV infection before renal transplantation and not via an infected graft or the surgery itself [117]. In the era of increasing emigration of people from high endemic area to industrialized countries, HBV infection will remain a persistent concern for public health authorities.

The impact of HBsAg seropositive status on patient survival after renal transplantation has been controversial [117]. Initial reports mostly focused on 5-year survival rates and generally failed to show a difference between HBsAg-positive and -negative renal transplant patients. In contrast, other studies of appropriate size and longer followup have found an unfavorable effect of HBsAg [116]. Mathurin and colleagues reported that 10-year patient survival was significantly higher in noninfected (80% ± 3%) than in HBsAg-positive recipients (55% ± 6%) or in recipients who were anti-hepatitis C virus (HCV) positive (65% ± 5%) [118]. Multivariate analysis demonstrated that HBsAg was an independent factor associated with inferior patient survival at 10 years (P b.0001). Fabrizi and colleagues, in a subsequent meta-analysis, showed that HBsAg seropositive status was an independent risk factor for death [119].

A more recent study by Matos and colleagues attempted to identify factors that increase liver fibrosis formation in renal transplanted patients with HBV infection [120]. They followed 55 patients for a mean time of  5 ± 4  years posttransplant. Liver biopsies of post-renal transplantation with advanced fibrosis were compared with those with mild fibrosis. Logistic regression analysis was applied to variables (age, sex, estimated time since infection, history of renal transplantation, donor type, ALT index, HBeAg, anti-HCV or quantitative HBV DNA, post-RT time, and HBV-HCV coinfection). Sixty-six percent of patients were on a cyclosporine-based regimen. The only independent factor associated with more advanced fibrosis was increased length of time after transplant. Immunosuppression postrenal transplantation may augment HBV replication by a variety of mechanisms including by diminishing activity of cytotoxic T lymphocytes. Azathioprine may stimulate intracellular HBV synthesis. Furthermore, calcineurin inhibitors (CNIs) may have direct effects on HBV replication [116]. Reactivation of HBV infection even in HBs Agseronegative individuals whose serological profile indicates remote, resolved HBV infection is recognized after transplantation with reappearance of HBsAg in serum [117]. Newly recognized HBV infection after renal transplantation can reflect de novo infection or reactivation of prior resolved infection. Some investigators observed in renal transplanted recipients after transplantation an increased replication with reappearance or increase in serum HBV DNA levels and HBeAg as well as reappearance of serum HBsAg following apparent clearance of prior HBV infection. Reactivation may cause necroinflammatory liver injury with biochemical dysfunction. Recent cases of reactivation of HBV after RT have been reported [121, 122], and fulminant hepatic failure due to HBV reactivation has been already noted after renal transplantation [123]. The 2 proposed theories for viral reactivation after RT are as follows: the first hypothesis include “occult HBV infection” in the RT candidate before transplant and viral replication of HBV under the influence of immunosuppression. Another theory is the loss of protective immunity and infection by a mutated strain. Naturally occurring mutations in the preC region and core promoter region are found in patients with long-standing chronic HBV infection [124]. Renal transplant recipients are at increased risk of developing a variety of cancers. Hepatocellular carcinoma is usually associated with viral hepatitis (HBV and HCV) but has been reported in postrenal transplanted recipients without overt evidence of viral hepatitis, cirrhosis, or metabolic liver disease [125].

Effective and safe vaccines that contain HBsAg, initially plasma derived and more recently manufactured by recombinant DNA technology, have been available since the early 1980s [126]. Vaccination against HBV is recommended for all susceptible patients on dialysis (i.e., those having anti-hepatitis B surface titers b10 IU/mL). Patients on maintenance dialysis have a suboptimal response (40%–50%) to HBV vaccine compared with the general population (N95% response). Several approaches have been tried to enhance response rates to HBV vaccine in these patients. These have included the administration of double or multiple doses of HBV vaccine, intradermal administration of vaccine, and vaccination with the simultaneous use of adjuvants (granulocyte macrophage-stimulating factor, thymopentin, erythropoietin, zinc supplements, etc.). Current recommendations for HBV vaccination in susceptible dialysis patients are administration of 4 double doses (40 *μ*g each dose) by intramuscular route at 0, 1, 2, and 6 months [126].

Evaluation of the donor includes testing with suspected HBV infection including HBsAg, HBsAb, and anti-HBc. The risk of HBV transmission to the renal transplanted recipient is a function of serologic status of both donor and recipient. A donor with HBsAb-positive serology due to vaccination will not transmit HBV infection. A donor with isolated anti-HBc-positive serology should have further serologic testing done to differentiate acute infection or remote exposure. If an IgM anticore is detected, the patient may have a recent acute HBV infection and should not be used in an HBV naive recipient without antiviral prophylaxis. Although livers harvested from isolated anti-HBc positive donors have a considerable risk of HBV transmission to recipients of liver grafts, kidneys from the same donors carry a low risk of transmission [127]. Kidneys from HBsAg-positive donors have a higher risk of transmitting HBV infection to their recipients, and the risk of transmission is greater if the donor status is HBsAg positive along with being HBeAg positive. HBsAb-positive recipients should not require prophylaxis [116]. On the other hand, organs from HBsAg-positive donors can be used in HBcAb-positive/HBsAb-positive recipients while requiring lamivudine prophylaxis (1 year). There are 6 licensed drugs available for the treatment of chronic HBV (interferon, lamivudine, adefovir, entecavir, telbivudine, and tenofovir). To date, interferon has not been promising in the management of HBV in renal transplanted recipients. There have been no studies regarding the use of telbivudine and tenofovir after renal transplantation. However, the management of HBV after renal transplantation has given encouraging results with lamivudine, adefovir, and entecavir [116].

The role of liver biopsy in the evaluation of RT candidates with HBsAg is important because it is difficult, on clinical grounds alone, to estimate the severity of liver disease in CKD population. Patients with established cirrhosis on liver biopsy are at risk for hepatic decompensation after isolated RT, and kidney transplantation alone is contraindicated. In patients with normal renal function, antiviral therapy has been reported to lead to regression of advanced histologic lesions.

Similar to hepatitis B, hepatitis C is a complex problem for the renal transplant recipient. Hepatitis C virus belongs to family *Flaviviridae*, genus Hepacivirus, species Hepatitis C virus Incidence rates in the chronic disease population have decreased, averaging 0.7 to 3 percent per year [30]. Recognition of hepatitis C in patients with chronic renal failure may be confounded by the limited sensitivity of serologic diagnosis in this population. All hepatitis C seronegative transplant candidates who have abnormal transaminases and/or risk factors for hepatitis C should undergo nucleic acid testing. Because of the increased risk of progressive liver disease following transplantation, patients with hepatitis C should undergo liver biopsy to exclude advanced liver disease, which in some cases may necessitate a combined liver-kidney transplant [128]. Hepatitis-C-positive patients usually have a marked rise in viral load with initiation of immunosuppression immediately after transplant. Chronic immunosuppression used in transplant recipients also leads to higher circulating levels of virus and intrahepatic virus due to the decreased T-cell response to the NS3 region of hepatitis C virus [30]. Consequently patients with hepatitis C are at increased risk for progressive liver disease and the development of cirrhosis following transplantation.

Because of the large demand for organs, consideration has been given to the use of hepatitis-C-positive donors. Transplant recipients may acquire hepatitis C through transplantation itself, which may lead to severe hepatitis. Although recipients of HCV positive kidneys have diminished patient and graft survival as compared with recipients of HCV-negative kidneys, survival may be improved when compared with survival on dialysis [129].

The problem of anti-HCV therapy is that interferon (IFN) has been associated with an increased risk of allograft rejection not only in the functioning grafts but also in the already failed grafts [130]. Therefore this may explain the major hesitancy to the use of IFNa in renal transplant recipients. It increases rejection possibly by cytokine gene expression, increased cell surface expression of HLA antigens, and enhanced function of natural killer cells, cytotoxic T cells, and monocytes. These immune-stimulant effects can result in enhanced allograft rejection—which may be irreversible—even in patients with stable grafts. So it has been indicated only in patients with fibrosing cholestatic hepatitis or other conditions in which the benefits of treatment outweigh the risk of allograft loss [131]. There are extremely limited data evaluating the efficacy and complications of IFNa therapy in the treatment of possible HCV-related cryoglobulinemia or de novo or recurrent glomerular disease in renal transplant recipients. Pageaux et al. [132] showed that treatment of HCV with IFN after transplantation might not be as risky as initially shown. Posttransplant monotherapies with ribavirin and/or amantadine have no apparent impact on HCV viremia or liver histology [133]. So the best strategy is to treat HCV infection in patients on dialysis before transplantation after excluding cirrhosis by liver biopsy and if present patients should be considered for combined kidney-liver transplantation [128, 132, 133]. Such strategy could prevent liver disease progression and even HCV-related morbidities such as glomerulonephritis and posttransplant diabetes mellitus.

## 9. Human Immunodeficiency Virus

Human immunodeficiency virus (HIV) belong to family *Retroviridae*, genus Lentivirus, species Human immunodeficiency virus. HIV infection has been a major global health problem for almost three decades. With the introduction of highly active antiretroviral therapy (HAART) in 1996, and the advent of effective prophylaxis and management of opportunistic infections, AIDS mortality has decreased markedly. In developed countries, this once fatal infection is now being treated as a chronic condition. As a result, rate of morbidity and mortality from other medical conditions leading to end-stage liver, kidney, and heart disease is steadily increasing in individuals with HIV. Renal diseases directly related to HIV infection include HIV-associated nephropathy (HIVAN), immune complex diseases, and thrombotic microangiopathy [134]. Although the widespread use of HAART has decreased the incidence of HIV-related renal disease, the overall prevalence of renal disease continues to increase among patients with HIV. The most aggressive HIV-related renal disease is HIVAN, which occurs in approximately 10% of patients with HIV [135]. These patients can progress to end-stage renal disease (ESRD) within weeks to months.

Recent studies confirm that outcome of renal transplantation in adequately selected HIV-infected patients receiving kidneys from HIV-negative donors is similar to that of HIV-negative RT recipients [136]. Main challenges in the clinical management of HIV-infected RT recipients are the pharmacologic interactions between immunosuppressive agents and some classes of antiretroviral drugs and a higher rate of acute rejection in comparison with HIV-negative RT recipients. Currently, organ transplantation from HIV-infected donors is an absolute contraindication in Western countries but its potential utility is under consideration [137] Recently, Muller et al. [138] reported the outcome of four HIV-infected RT recipients who received their grafts from two HIV-infected donors in South Africa, being the first clinical experience published involving this type of transplants. After 12 months of followup, all recipients had a functioning renal allograft with a good renal function, and HIV infection was controlled under different antiretroviral regimens. South Africa is a country with a high HIV prevalence in the general population, and HIV infection is an absolute exclusion criterion for access to dialysis or RT. Muller et al. [138] suggested that the use of HIV-infected donors would increase the donor pool, providing renal allografts to HIV-infected patients otherwise sentenced to death as a consequence of end-stage renal disease. Deceased HIV-infected patients represent a potential of approximately 500–600 donors per year for HIV-infected transplant candidates. In the current era of HIV management, a legal ban on the use of these organs seems unwarranted and likely harmful [139].

HIV-infected patients receiving renal transplants may be at higher risk of acute rejection (up to 25%) and the optimal management of immunosuppression in HIV-infected individuals remains unknown [140]. Treatment of rejection with cytolytic agents such as thymoglobulin may result in prolonged depression of CD4 counts and significant infection-related morbidity [141]. The risks of antilymphocyte therapy should be balanced with the risks for rejection in HIV-infected recipients.

## 10. Respiratory Viruses

Various viruses can cause respiratory disease in the renal transplant population, including adenovirus, respiratory syncytial virus, influenza, parainfluenza, human metapneumovirus, rhinovirus, and coronavirus [30]. These viruses can lead to upper respiratory tract disease, as well as bronchitis, pneumonitis, and pneumonia. Adenovirus can cause a multitude of complications including gastroenteritis, cystitis, and necrotizing hepatitis in addition to respiratory illness. In immunocompromised patients, adenovirus infections tend to be more prolonged, more severe, and sometimes fatal. They may occur due to endogenous reactivation or primary infection. Coinfection with more than one adenovirus serotype per clinical event was more frequent in immunocompromised patients (30%) than in immunocompetent patients (5%) [142]. Clinical manifestations in immunocompromised patients include pneumonia, hepatitis, hemorrhagic cystitis, colitis, pancreatitis, meningoencephalitis, and disseminated disease, depending on the underlying disease, affected organ system, patient age, and virus serotype. In the renal transplant recipient, adenovirus can also cause nephritis, which presents with fever, renal dysfunction and liver function test abnormalities [143, 144]. Infection with respiratory viruses may also be associated with rejection. Severity of disease is greatest in those who have been more recently transplanted or are more immunosuppressed.

Clinicians and pathologists should be vigilant and aware of AV nephritis in renal allografts. Diagnosis can be difficult. Few laboratories perform immunohistochemistry for AV. Multiple diagnostic modalities, including immunohistochemistry of biopsy material, serology, viral cultures, and viral gene PCR may be required to make the diagnosis. Pathological clues to the diagnosis of AV infection include gross haematuria, often severe, with haemorrhagic cystitis and fever with or without pneumonitis. On renal biopsy, the presence of tubulointerstitial necrosis, haemorrhage, and atypical “smudged” epithelial cells can be helpful in recognizing the lesion. Although most AV infections occur within weeks or months of transplantation due to activation of a latent infection, AV infection can also uncommonly occur years later. This may be due to a de novo AV infection [144].

Prevention involves avoidance of other individuals who have signs or symptoms of infection, hand hygiene, and use of droplet precautions for those suspected of having infection. Influenza vaccine should be administered before transplant and every year after transplant, although administration should not be given in the early posttransplant period because of especially reduced vaccine responses [30]. Because the vaccine may be insufficiently protective in the patient after transplant, the influenza vaccine should also be administered to the patient's family members and to health care providers to decrease possible risk of transmission. Intranasal influenza vaccine should not be used, as it is utilizes live virus [20]. Morelon et al. [145] suggest that intradermal influenza vaccination may offer enhanced immunogenicity and protection in persons who do not respond well to conventional trivalent inactivated influenza vaccine. Further studies should be conducted in immunocompromised populations to validate the trends for higher efficacy of ID versus conventional route of immunization against influenza.

Treatment of respiratory viral infections involves supportive care and, in some cases, the use of antiviral medications. Influenza can be treated with oseltamivir or zanamavir, which will treat both influenza A and B. Amantadine is not recommended because it treats influenza A only and increasing rates of resistance have been seen in influenza A. Ribavirin is approved to treat lower respiratory infection with respiratory syncytial virus; however actual clinical efficacy has not been proven. Severe adenovirus infections are usually treated with reduction of immunosuppression. Anecdotal reports suggest that cidofovir may have activity against adenovirus, but its use must be balanced with the associated risk of nephrotoxicity [146, 147].

## 11. West Nile Virus

West Nile virus (WNV) belong to family *Flaviviridae*, genus Flavivirus, species West Nile virus. Since its isolation in Uganda in 1937, WNV has been responsible for thousands of cases of morbidity and mortality in birds, horses, and humans. Historically, epidemics were localized to Europe, Africa, the Middle East, and parts of Asia, and primarily caused a mild febrile illness in humans. However, in the late 1990s, the virus became more virulent and expanded its geographical range to North America. In humans, the clinical presentation ranges from asymptomatic (approximately 80% of infections) to encephalitis/paralysis and death (less than 1% of infections) [148].

WNV was recently transmitted from one donor (likely related to a blood transfusion) to multiple recipients [149]. Transplant recipients are at higher risk than the general population for meningoencephalitis after exposure. In WNV endemic regions, donors should be tested for WNV. Virus from their donors have increased morbidity and mortality [150, 151]. Recipients who acquire West Nile later in the transplant course have more variable outcomes. To prevent infection, seasonal screening should be considered for donors before transplant by serologic and/or nucleic acid testing. Treatment for West Nile in transplant recipients has not been standardized but should include a reduction in immunosuppression along with supportive care [151]. Whether there is a role for hyperimmune globulin in transplant recipients is currently unknown. Viral infections in renal transplant patients continue to have significant impact on patient outcomes. Although preventive measures have improved, the impact of newer immunosuppressive strategies continues to promote the development of severe viral infections [20]. The presence of new and emerging viral infections that may be transmitted by transplantation, such as West Nile virus, will likely present many future challenges for physician treating transplant recipients.

## Figures and Tables

**Table 1:** Common viral pathogens in renal transplant recipients, time of their presentation, prevalence, prevention and available cures.

Common viral pathogens in renal transplant recipients	Time of their presentation	Prevalence in renal transplant population	Prevention	Therapy and available cures
Cytomegalovirus	Months 3–12 Months 1–3 (in absence of prophylaxis) Beyond 1 year (in setting of extended prophylaxis)	8–32%	Serologic screening for antibody donor and recipient, universal prophylaxis include acyclovir, ganciclovir, valacyclovir, valganciclovir, and immune globulin preparations	Combination of immunomodulation, antiviral therapy (intravenous ganciclovir, valganciclovir, foscarnet, cidofovir, and leflunomide) and reduction of immunosuppression
Herpes simplex	First Month, Months 1–3	approximately 53%	Serologic screening for antibody donor and recipient	Acyclovir
Varicella zoster	Months 1–6	4–12%	Serologic screening for antibody donor and recipient, live attenuated varicella vaccine	Ganciclovir, valacyclovir, acyclovir
Epstein-Barr virus	Months 1–12	1–3%	EBV load testing is commonly used	Reduction of immunosuppression, infusing donor lymphocytes, infusing EBV-specific cytotoxic T and infusing anti-CD20 monoclonal antibody
Human herpesvirus-6	First Month, Months 1–3	No data	Serologic screening for antibody donor and recipient	Reduction of immunosuppression, ganciclovir, cidofovir, foscarnet
Human herpesvirus-7	First Month and Months 1–3	No data	Serologic screening for antibody donor and recipient	Reduction of immunosuppression, ganciclovir, cidofovir, foscarnet
Human herpesvirus-8	Months 6–12 and beyond 1 year	0.2–5%	Optional screening measures	Reduction of immunosuppression, rapamycin
Polyomavirus BK/JC	Months 3–12 and beyond 1 year	1%–10%	Quantitative nucleic acid-based viral load assay of urine or blood, urine cytology, polymerase chain reaction	Reduction of immunosuppression, cidofovir, leflunomide, quinolones, and intravenous immunoglobulin
Hepatitis B and C (acquisition of new infection)	Months 1–12 and beyond 1 year	No data	Serologic screening for antibody donor and recipient	Reduction of immunosuppression lamivudine, adefovir, and entecavir
Hepatitis B and C (reactivation)	First Month, Months 1–3	2–10%	Serologic screening for antibody donor and recipient	Interferon (associated with an increased risk of allograft rejection)
Human immunodeficiency virus	No data	No data	Serologic screening for antibody donor and recipient	The optimal management of immunosuppression in HIV infected individuals remains unknow
Adenovirus, respiratory, syncytial virus, influenza, parainfluenza, human metapneumovirus, rhinovirus, and coronavirus	Months 1–12 and beyond 1 year	No data	Influenza vaccine	Oseltamivir, zanamavir, ribavirin, cidofovir
West Nile virus (acquisition from transplant)	First Month	No data	Optional Screening Measures	Reduction of immunosuppression
West Nile virus (acquired after transplant)	Months 1–12 and beyond 1 year	No data	Optional Screening Measures	Reduction of immunosuppression
